# Selective JAK 1 inhibition with upadacitinib as a potential treatment for coexistent severe atopic dermatitis and alopecia areata^⋆^^⋆⋆^

**DOI:** 10.1016/j.abd.2023.07.003

**Published:** 2024-02-24

**Authors:** Guilherme Muzy

**Affiliations:** Clínica Muzy, São Paulo, SP, Brazil

Dear Editor,

Atopic dermatitis (AD) is a chronic inflammatory skin disease characterized by pruritus, erythema, and scaling. It affects approximately 15%‒30% of children and 2%‒10% of adults worldwide, making it a prevalent condition that can significantly impact the quality of life of affected individuals.^1^ On the other hand, alopecia areata (AA) is an autoimmune disease that affects hair follicles, leading to hair loss in patches on the scalp, face, or body. The estimated lifetime risk of developing AA is 2.1%.^2^ Although AD and AA are two distinct diseases, they have been reported to coexist in some patients, with the prevalence of AA in patients with AD ranging from 0.3% to 6.8%.^3^

We present a case of a 27-year-old male patient with a 20-year history of severe AD and an 8 month history of a single patch of AA. No history of other type 2 mediated diseases such as asthma or allergic conjunctivitis. Despite previous treatments for AD, including phototherapy and methotrexate, the patient's skin disease remained uncontrolled. Before beginning therapy with Upadacitinib, a selective Janus kinase (JAK) 1 inhibitor, the patient had a SCORing Atopic Dermatitis (SCORAD) score of 52.15, an Investigator Global Assessment (IGA) score of 4, a pruritus Numeric Rating Scale (NRS) score of 9, a Severity of Alopecia Tool (SALT) score of 13, and a Dermatology Life Quality Index (DLQI) score of 25 (Figure 1, Figure 2).

**Figure 1:**
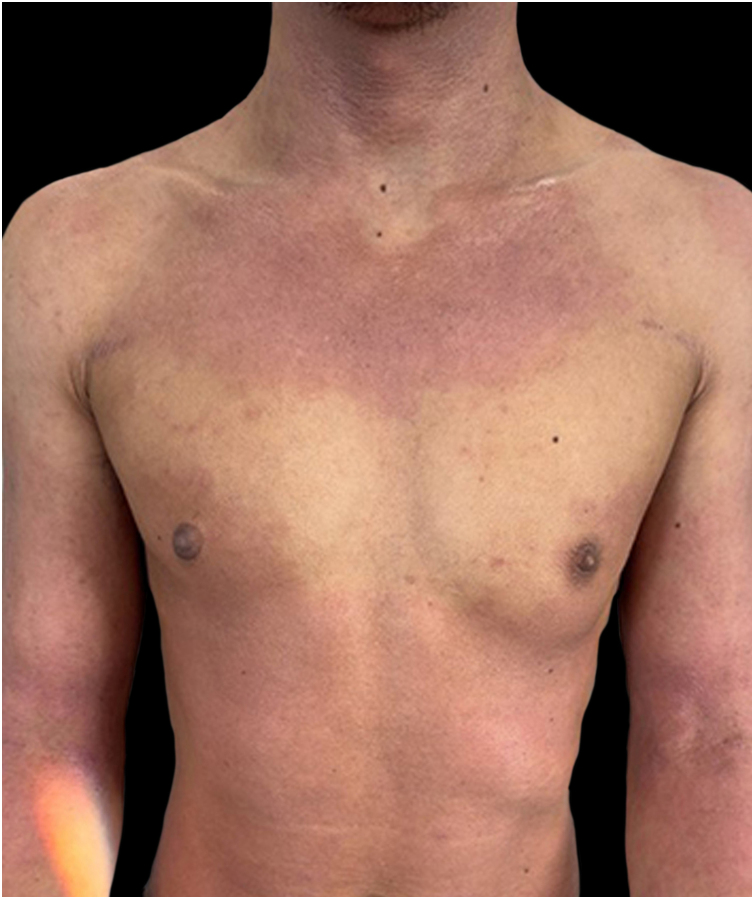
Severe, widespread erythematous patches of atopic dermatitis, with different degrees of lichenification.

**Figure 2:**
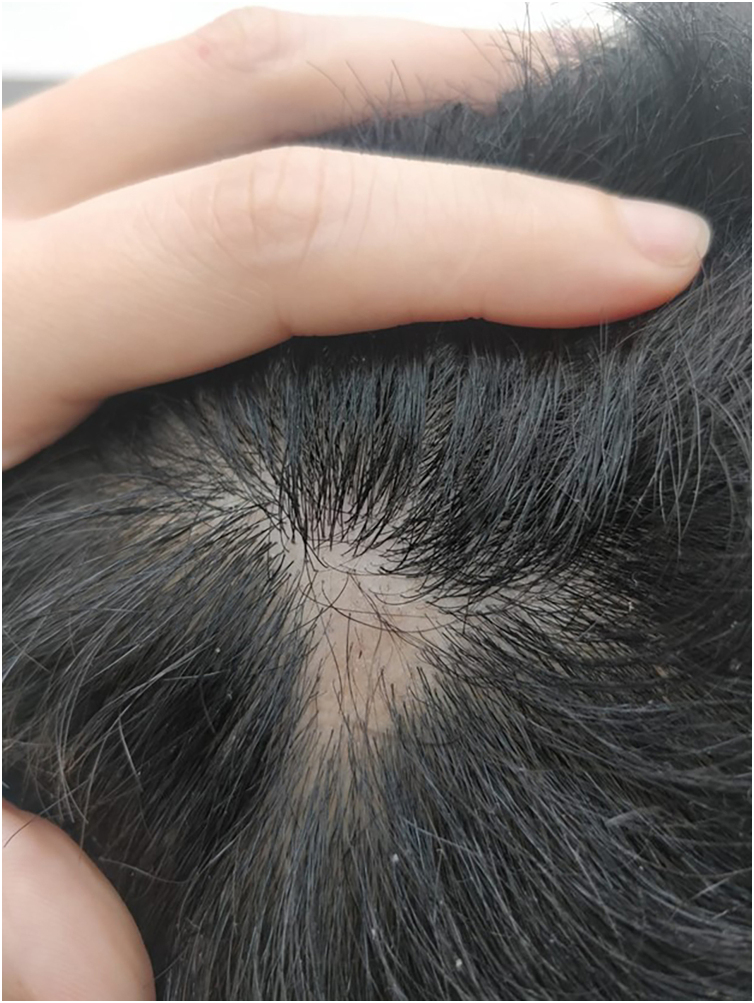
Small localized patch of alopecia areata. Note the “exclamation dot hair” in the center.

Upadacitinib has been shown to be effective in treating moderate-to-severe AD in adult patients in randomized, double-blind, placebo-controlled, phase 3 trials.^4^ Furthermore, recent studies have suggested that JAK inhibition may also be effective in treating AA by suppressing the activity of cytotoxic T lymphocytes, which play a crucial role in the pathogenesis of the disease,^5^ and also by reducing IFN-gamma signaling through selective inhibition of JAK 1.^6^, ^7^

Given the patient's refractory AD and coexisting AA, Upadacitinib 15 mg daily was initiated as a therapeutic option. After four weeks of treatment, the patient's pruritus NRS score decreased to 0, and after eight weeks, his SCORAD score decreased to 7.1, his IGA score decreased to 0, and his DLQI score decreased to 2. Notably, the patient also reported significant improvement in his AA, with regrowth of hair in previously affected areas and his SALT score decreased to 0 at 16 weeks (Figure 3, Figure 4).

**Figure 3:**
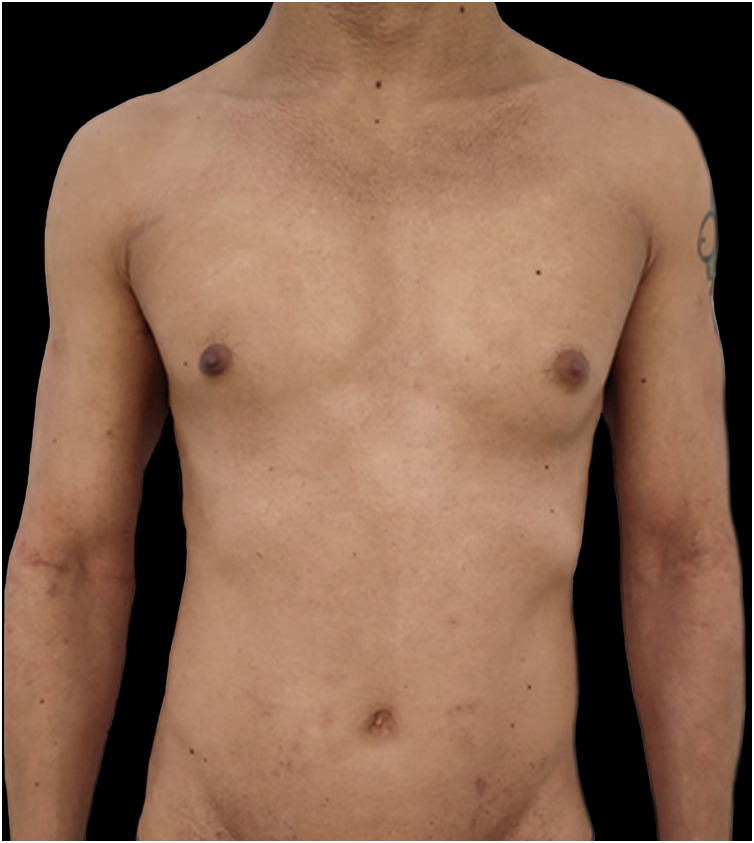
Complete skin clearance after 16-weeks of upadacitinib 15 mg daily. Note a few residual hyperchromic macules.

**Figure 4:**
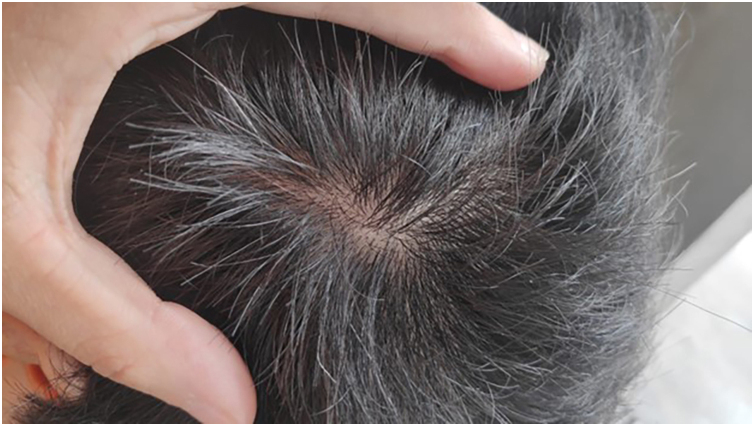
Resolution of patch of alopecia areata after 16-weeks of Upadacitnib 15 mg daily.

In conclusion, the coexistence of AD and AA is common, with a significant effect on the associated prevalence of AA between patients with AD and controls (relative risk of 5.78, 95% Confidence Interval, 3.82‒8.73).^8^ This case highlights the potential of Upadacitinib 15 mg daily as a therapeutic option for patients with severe AD and AA due to the effects of selective JAK 1 inhibition in the immunopathogenesis of both diseases. The efficacy of Upadacitinib in managing both diseases is sustained at the 8-month follow-up. Further research is needed to investigate the long-term safety and efficacy of Upadacitinib in this patient population.

## Financial support

The work presented has not received any financial support from the pharmaceutical industry or any other commercial source, except as described below, and i have received speaker fees from the following companies: AbbVie, Eli Lilly, Janssen, Sanofi, and Pfizer.

## Author' contributions

Guilherme Muzy: Approval of the final version of the manuscript, critical literature review, data collection, analysis, and interpretation; effective participation in research orientation, intellectual participation in propaedeutic and/or therapeutic management of studied cases, manuscript critical review, preparation, and writing of the manuscript, statistical analysis and study conception and planning.

## Conflicts of interest

None declared.
